# Lack of health insurance is associated with delays in PrEP initiation among young black men who have sex with men in Atlanta, US: a longitudinal cohort study

**DOI:** 10.1002/jia2.25399

**Published:** 2019-10-08

**Authors:** David P Serota, Eli S Rosenberg, Annie L Thorne, Patrick S Sullivan, Colleen F Kelley

**Affiliations:** ^1^ Department of Medicine, Division of Infectious Diseases Emory University School of Medicine Atlanta GA United States; ^2^ Department of Epidemiology and Biostatistics School of Public Health SUNY University of Albany Rensselaer NY United States; ^3^ Department of Behavioral Science and Health Education Emory University Rollins School of Public Health Atlanta GA United States; ^4^ Department of Epidemiology Emory University Rollins School of Public Health Atlanta GA United States

**Keywords:** PrEP, health insurance, young black men who have sex with men, health disparity, sexually transmitted infections, adolescent health

## Abstract

**Introduction:**

Delays between receiving a PrEP prescription and taking a first dose increase the risk of HIV infection. This is especially relevant in populations with high HIV incidence, such as young black men who have sex with men (YBMSM) in the United States. Additionally, YBMSM have relatively low levels of health insurance. We investigated whether lack of health insurance and reliance on PrEP funding through the manufacturer assistance programme (MAP) leads to delays in initiation of PrEP.

**Methods:**

HIV‐negative YBMSM were offered PrEP as part of a prospective cohort. Enrolment began in June 2015 with follow‐up through February 2019. Interested participants attended a PrEP clinician visit and received a prescription. Those with health insurance received a copay assistance card; those without insurance accessed PrEP using the MAP. The primary outcome was the days between prescription and initiation. The effect of insurance status on this delay was modelled using a Cox proportional hazards model.

**Results and Discussion:**

The median delay between receipt of a PrEP prescription and taking a first dose was 12 days (IQR 3 to 32). Compared to uninsured participants, the adjusted hazard ratio for PrEP initiation for those with insurance was 2.72 (95% CI 1.82 to 4.06). The adjusted median time to initiation for insured participants was 5 days versus 21 days for those without insurance (*p *<* *0.0001). Older age and STI diagnosis were also associated with faster PrEP initiation. Despite equivalent access to PrEP provided by the study, YBMSM without insurance had longer delays in initiation after receipt of a prescription. Overall, the observed delay in PrEP initiation increases the chances of HIV infection and the possibility of PrEP initiation after undetected seroconversion.

**Conclusions:**

The extended time period between PrEP prescription and taking a first dose increases the risk of HIV transmission. Younger YBMSM and those without health insurance had longer delays in PrEP initiation. Immediate PrEP initiation programmes could decrease the likelihood of this occurrence and mitigate the disparity in initiation between those with and without health insurance. Clinical Trial Number: NCT02503618.

## 1 Introduction

HIV incidence in the United States is highest among young black men who have sex with men (YBMSM), yet uptake of HIV pre‐exposure prophylaxis (PrEP) remains low in this population (PrEP) 1, 2, 3. Among the steps of the PrEP continuum of care, successful implementation requires access to PrEP and expedient initiation following confirmation of HIV‐negative status 4. Any delay in PrEP initiation in a high incidence population has the potential to lead to preventable HIV infections. The United States (US) Center for Disease Control and Prevention (CDC) PrEP guidelines recommend initiation of PrEP within 7 days of a negative HIV test 5; however, the time delay between prescription and taking a first dose of PrEP in clinical settings in not well described. In the event that someone acquires HIV prior to their first dose of PrEP but then goes on to initiate PrEP, diagnosis can be delayed due to unusual patterns in seroconversion and drug resistance can develop 6.

We previously reported on the systems barriers faced by YBMSM in a cohort of YBMSM in Atlanta, Georgia (GA), United States to obtaining PrEP, and anecdotally noted delays in initiation due to health insurance prior authorization, lack of coverage and requirements of enrolment in drug manufacturer assistance programmes (MAPs) 7, 8. Here we sought to quantify in‐depth the relationship between health insurance status and delays from PrEP prescription receipt to PrEP initiation. We also evaluated for other potential predictors of delays between PrEP prescription and taking a first dose.

## 2 Methods

### 2.1 Study design

The EleMENt study was a longitudinal cohort of sexually active YBMSM (18 to 29 years) in Atlanta, Georgia designed to evaluate the relationship between substance use and HIV/sexually transmitted infections (STI) (NCT02503618). Enrolment began in June 2015 with follow‐up through February 2019. HIV‐negative participants were followed prospectively over 24 months. At each visit, participants were offered enrolment in an optional PrEP programme, as described elsewhere 8.

Interested participants attended a PrEP clinician visit to determine medical eligibility and obtain recommended laboratory testing 5. Insured participants were given a prescription for daily tenofovir disoproxil fumarate/emtricitabine (TDF/FTC) and offered a copay assistance card. Uninsured participants completed manufacturer assistance programme (MAP) forms before or during the clinician visit, provided accompanying required documentation, and were given a prescription. Participants who did not submit documentation for the MAP at the time of their visit were contacted regularly with reminders to submit documents. Study staff then submitted MAP forms along with other required documents within one business day. Study staff worked with the participants to submit acceptable alternatives as needed. Once forms were submitted, staff followed up with the drug company daily to check on processing. Once approved, staff provided the participant with approval codes to be given to the pharmacy and instructions for how to use the codes. Study staff were available via phone if participants had problems picking up prescriptions.

Participants were contacted regularly after their PrEP clinician visit to ensure they had successfully obtained TDF/FTC and to provide assistance if they had not. Approximately one month after the clinician visit, staff called each participant to formally assess initiation and side effects and to provide adherence counselling. Each participant was asked to estimate the date of first PrEP dose. If a participant had not initiated PrEP at the one‐month call, staff helped troubleshoot barriers to initiation and called at least monthly to assess for initiation.

### 2.2 Insurance, covariates and outcome

The exposure of interest was health insurance status at the time of PrEP clinician visit, with all other variables collected at the baseline study visit. Insurance could be private commercial health insurance or government programmes (e.g. Medicaid, Medicare, Tricare). All uninsured participants pursued access to PrEP through the MAP. Other covariates identified in previous analyses as contributing to PrEP uptake and/or persistence including cannabis use, STIs and self‐efficacy were assessed 8, 9, 10. Cannabis use was defined by a composite of self‐reported use in the prior 6 months *or* a positive urine drug screen. STI history classified per self‐report in the last 12 months or a positive test for syphilis, gonorrhoea or chlamydia. Depression symptoms (moderate to severe), self‐efficacy and discrimination were defined using validated scales 11, 12, 13. The outcome was number of days between receipt of a PrEP prescription—which occurred at the “PrEP clinician visit”—and self‐report of taking a first dose of PrEP (PrEP initiation).

### 2.3 Analyses

We summarized demographic factors, the above covariates and insurance status descriptively for all participants who attended a PrEP clinician visit, stratified by whether they ultimately initiated PrEP before study completion. Subsequent analyses evaluating the delay in PrEP initiation were restricted to those who initiated PrEP. A Cox proportional hazards model was used to estimate the adjusted association between insurance status and time to PrEP initiation. Potential confounders were chosen *a priori* based on hypothesized causal pathways and prior analyses from this cohort 8, 9, 10. All covariates were included in the final model. The model was used to estimate the adjusted hazard ratio and cumulative incidence functions for insurance status, balanced across covariates using inverse weighting 14. Plots of ln(ln[S(t)]) affirmed no violation of the proportional hazard assumption.

### 2.4 Ethics statement

All participants enrolled on a voluntary basis and completed a written informed consent. Study staff verbally confirmed understanding. The EleMENt study was reviewed and approved by the institutional review board of Emory University.

## 3 Results and discussion

Of the 298 YBMSM enrolled in EleMENt, 154 (52%) attended a PrEP clinician visit and received a PrEP prescription. At the end of follow‐up, 131 (44%) reported taking a first dose of PrEP. Table 1 presents characteristics of the 154 participants who attended a PrEP clinician visit, stratified by PrEP initiation. At the time of the PrEP clinician visit, 45% were uninsured. All but one of the insured participants had private health insurance, with one having Tricare.

**Table 1:** Baseline characteristics of participants prescribed PrEP in a cohort of young black men who have sex with men (N = 154)

Variable	No PrEP initiation (N = 23), N (%)	PrEP initiation (N = 131), N (%)	All participants prescribed PrEP (N = 154), N (%)
Age, median (IQR)			
18 to 21	6 (26)	19 (15)	25 (16)
22 to 25	12 (52)	58 (44)	70 (45)
26 to 29	5 (22)	54 (41)	59 (38)
≥High school	15 (65)	101 (78)	116 (76)
Unemployed	2 (9)	10 (8)	12 (8)
Health insurance^a^			
None	9 (39)	61 (47)	70 (45)
Private/Public	14 (61)	70 (53)	84 (55)
Marijuana use	16 (70)	81 (62)	97 (63)
Depression	5 (23)	22 (17)	27 (18)
Low self‐efficacy	7 (32)	21 (16)	28 (19)
Discrimination	11 (50)	50 (40)	61 (41)
STI past 12 months	9 (39)	66 (50)	75 (49)

IQR, interquartile range; PrEP, pre‐exposure prophylaxis; STI, sexually transmitted infection.

a Health insurance status assessed at time of PrEP prescription rather than baseline study visit.

**Figure 1:**
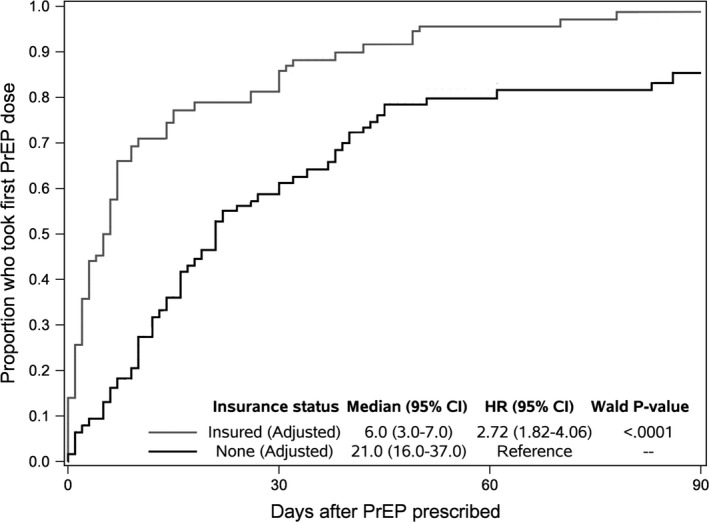
Cox adjusted cumulative incidence plot for time to PrEP initiation after prescription.Plot shows the Cox‐adjusted 1‐survival function for time to PrEP initiation after prescription. The black curve shows participants with no health insurance and the grey line represents participants with health insurance. The plot is adjusted for all variables in Table 2. The plot is right‐truncated at 90 days after PrEP prescription, though eventually 100% took a first dose of PrEP.

Among the 131 participants who initiated PrEP, the median time between PrEP clinician visit and PrEP initiation was 12 days (IQR 3 to 32 days). Results (Table 2) showed that compared to 18‐ to 21‐year olds, 22‐ to 25‐year olds initiated PrEP 1.93 times faster, and 26‐ to 29‐year olds had 2.56 times the rate of initiation. Those with health insurance had a 1.72 times higher rate (aHR) of initiation, compared to those with no insurance (who relied on the MAP). Per the adjusted cumulative incidence plot for the time from PrEP prescription to initiation, the adjusted median time to initiation was 5 days (95% CI 3 to 7) for insured participants, compared to 21 days (95% CI 16 to 37) for uninsured participants (Figure 1).

**Table 2:** Cox proportional hazard model for time delay between PrEP prescription and PrEP initiation

Variable	HR	95% CI		*p*‐value	aHR	95% CI		*p*‐value
Age								
18 to 21	Ref				Ref			
22 to 25	1.61	0.94	2.77	0.08	1.93	1.06	3.52	0.03
26 to 29	1.83	1.06	3.19	0.03	2.56	1.35	4.83	0.004
Education level	1.27	0.84	1.92	0.27	1.02	0.64	1.62	0.94
Unemployed	0.82	0.43	1.56	0.55	0.53	0.26	1.08	0.08
Health insurance								
None	Ref				Ref			
Private/Public	2.11	1.48	3.02	<0.0001	2.72	1.82	4.06	<0.0001
Marijuana use	0.93	0.65	1.33	0.71	1.04	0.70	1.55	0.83
Depression	0.96	0.61	1.53	0.87	1.46	0.82	2.61	0.20
Low self‐efficacy	0.91	0.57	1.45	0.69	0.66	0.38	1.14	0.14
Discrimination	0.81	0.56	1.16	0.25	0.86	0.56	1.31	0.47
STI past 12 months	1.32	0.91	1.90	0.14	1.55	1.05	2.30	0.03

aHR, adjusted hazard ratio; CI, confidence interval; HR, hazard ratio; PrEP, pre‐exposure prophylaxis; STI, sexually transmitted infection.

In this cohort of YBMSM with access to free PrEP clinical services and PrEP navigation, there was a median 12‐day delay between receipt of a prescription for PrEP and taking a first dose of medication. Lack of health insurance and younger age were independently associated with longer delays in initiation, while history of STI was associated with faster initiation. Because HIV incidence is high among YBMSM in Atlanta, this delay in initiation has potential to lead to HIV infections that would have been averted with faster PrEP initiation 7, 15. Because most PrEP clinical trials and demonstration projects have directly provided medication outside of the insurance and healthcare systems, the delays in initiation we have documented in a community‐based setting have not been well described.

We previously identified lack of health insurance as a risk factor for acquiring HIV infection among black MSM in Atlanta 15, and those who choose to initiate PrEP have a higher prevalence of sexual risk behaviours 16, 17. Thus, our finding that PrEP‐interested YBMSM without insurance have the longest delays between receipt of a PrEP prescription and PrEP initiation is especially concerning. Encouragingly, those with highest HIV risk in the cohort—history of STI—had faster initiation. In the current US healthcare climate, MAPs are crucial to providing PrEP to those without health insurance, particularly in states such as Georgia which have not expanded Medicaid following passage of the Affordable Care Act. However, our experience shows that in comparison to insured patients, delays associated with MAPs have the potential to lead to HIV infections that could have been prevented with more expedient PrEP initiation.

A study modelling the burden of financial barriers to PrEP estimated that only 7% of those with an indication for PrEP would need financial assistance outside of health insurance and that most of those would be able to obtain funding using the MAP 18. In contrast, in our study, 47% of YBMSM were uninsured and required use of the MAP to access PrEP. Thus, while the estimated coverage gaps may seem trivial at first glance, they are not distributed equally over the population, and we show that reliance on the MAP is high among key, high‐incidence populations, such as YBMSM in the southeast United States. Additionally, the United States Centers for Disease Control and Prevention (CDC) guideline indications for PrEP are insensitive for HIV risk among YBMSM, which further increases the likely pool of uninsured candidates for PrEP 19.

Some participants had difficulty obtaining documents for the MAP or required multiple reminders to return the documents. Despite repetitive contact attempts, participant responses to phone calls and text messages were sometimes delayed. Anecdotally, the actual pharmaceutical company processing time was short once they received a complete application. Nonetheless, utilization of the MAP may delay PrEP initiation through the requirement for multiple points of contact with patients between the time of prescription and when the first dose is taken. More recent changes to the Gilead MAP may mitigate some of these delays, but were not reflected in the study period.

Rapid or immediate initiation of antiretroviral therapy for patients with HIV has been associated with increased retention in care and more rapid achievement of undetectable viral load 20, 21. One successful model of immediate PrEP initiation in New York City sexual health clinics provides a first 30‐day supply of PrEP to patients on the day of their visit, after negative rapid HIV testing 22. This 30‐day buffer could mitigate the delay we observed among participants relying on MAPs. In order to minimize these potential “PrEP systems failures” 7, further resources should be directed specifically to ensuring rapid access to PrEP at the time of HIV testing.

This study is limited by the potential for residual confounding between insurance status and the delay in PrEP initiation. It is possible that there are fundamental differences between participants with and without insurance that led to differential speeds of PrEP initiation and that these differences were not fully addressed by statistical modelling. PrEP initiation was a self‐reported date, which is prone to recall bias. Any conclusions that PrEP use translates into reduced HIV incidence must be tempered by the fact that PrEP use was self‐reported. The best available evidence showing that PrEP use leads to fewer HIV infections relies on more robust assessments of PrEP use. Results may not be generalizable to other populations (non‐YBMSM). Although our programme was closer to real‐world clinical practice than some PrEP clinical trials, we had grant funding to supply free PrEP clinical services (including lab testing) and provided PrEP navigation from study staff. Delays in PrEP initiation might have been longer without this PrEP navigation resource. Finally, study enrolment ensured that all participants received education and access to PrEP services, both of which remain barriers to PrEP expansion in the United States.

## 4 Conclusions

In conclusion, we demonstrated prolonged delays between receipt of PrEP prescription and initiation of TDF/FTC in a key population in a US city with high HIV incidence. Although uninsured participants theoretically had equal access to medication through the MAP, lack of insurance was strongly associated with longer delays in initiation. Interventions aimed at expediting PrEP uptake regardless of insurance status are needed.

## Competing interests

Dr. Sullivan reports grants from Gilead, National Institutes of Health (NIH), and CDC during the conduct of the study. Dr. Serota reports grants from National Center for Advancing Translational Sciences during the conduct of the study. Dr. Rosenberg reports grants from NIH and CDC during the conduct of the study. Ms. Thorne has nothing to disclose. Dr. Kelley reports grants from Gilead Sciences, NIH, and CDC during the conduct of the study.

## Authors’ contributions

DPS performed data analysis and wrote the initial draft of the manuscript. ALT, ESR, CFK and PSS all assisted DPS the design of the analysis, interpretation of the results and provided substantial edits to the text of the manuscript.
